# Psychometric properties of a standardized protocol of muscle strength assessment by hand-held dynamometry in healthy adults: a reliability study

**DOI:** 10.1186/s12891-023-06400-2

**Published:** 2023-04-14

**Authors:** Marika Morin, Luc J. Hébert, Marc Perron, Émilie Petitclerc, Shanna-Rose Lake, Elise Duchesne

**Affiliations:** 1 Department of Health Sciences, Université du Québec à Chicoutimi, 555 Bd de l’Université, Chicoutimi, QC G7H 2B1 Canada; 2 Department of Rehabilitation, and Department of Radiology and Nuclear Medicine, Faculty of Medicine, Université Laval, Quebec City, QC Canada; 3 Interdisciplinary Research Group On Neuromuscular Diseases (GRIMN), Integrated University Center of Health and Social Services of Saguenay–Lac-St-Jean, Jonquiere, Canada; 4 Interdisciplinary Research Centre for Rehabilitation and Social Integration (CIRRIS), Integrated University Center of Health and Social Services of the Capitale-Nationale, Quebec City, QC Canada; 5 Intersectoral Center for Sustainable Health, Université du Québec À Chicoutimi, Chicoutimi, QC Canada; 6 Research Center of Charles-Le Moyne (CRCLM), Sherbrooke, QC Canada

**Keywords:** Hand-held dynamometry, Muscle strength, Psychometric properties, Quantitative evaluation, Reliability, Standard error of measurement, Minimal detectable change

## Abstract

**Background:**

Maximal isometric muscle strength (MIMS) assessment is a key component of physiotherapists’ work. Hand-held dynamometry (HHD) is a simple and quick method to obtain quantified MIMS values that have been shown to be valid, reliable, and more responsive than manual muscle testing. However, the lack of MIMS reference values for several muscle groups in healthy adults with well-known psychometric properties limits the use and the interpretation of these measures obtained with HHD in clinic.

**Objective:**

To determine the intra- and inter-rater reliability, standard error of measurement (SEM) and minimal detectable change (MDC) of MIMS torque values obtained with HHD.

**Methods:**

Intra and Inter-rater Reliability Study. The MIMS torque of 17 muscle groups was assessed by two independent raters at three different times in 30 healthy adults using a standardized HHD protocol using the MEDup™ (Atlas Medic, Québec, Canada). Participants were excluded if they presented any of the following criteria: 1) participation in sport at a competitive level; 2) degenerative or neuromusculoskeletal disease that could affect torque measurements; 3) traumatic experience or disease in the previous years that could affect their muscle function; and 4) use of medication that could impact muscle strength (e.g., muscle relaxants, analgesics, opioids) at the time of the evaluation. Intra- and inter-rater reliability were determined using two-way mixed (intra) and random effects (inter) absolute agreement intraclass correlation coefficients (ICC: 95% confidence interval) models. SEM and MDC were calculated from these data.

**Results:**

Intra- and inter-rater reliability were excellent with ICC (95% confidence interval) varying from 0.90 to 0.99 (0.85–0.99) and 0.89 to 0.99 (0.55–0.995), respectively. Absolute SEM and MDC for intra-rater reliability ranged from 0.14 to 3.20 Nm and 0.38 to 8.87 Nm, respectively, and from 0.17 to 5.80 Nm and 0.47 to 16.06 Nm for inter-rater reliability, respectively.

**Conclusions:**

The excellent reliability obtained in this study suggest that the use of such a standardized HHD protocol is a method of choice for MIMS torque measurements in both clinical and research settings. And the identification of the now known metrological qualities of such a protocol should encourage and promote the optimal use of manual dynamometry.

**Supplementary Information:**

The online version contains supplementary material available at 10.1186/s12891-023-06400-2.

## Introduction

Muscle strength is a central component of function [1–4]. Deterioration in muscle strength below critical thresholds can have a significant impact on an individual's ability to accomplish activities of daily living [2, 3] and locomotion [5–7]. Physiotherapists need to adequately measure the magnitude of muscle weaknesses, as they will guide the clinical management of a given condition [8].

Different methods exist to measure maximal isometric muscle strength (MIMS), but present characteristics limiting their usefulness in clinical decision-making. The isokinetic dynamometer, for example, is the gold standard for measuring muscle strength [9], but it is costly and requires a large space and considerable user training, limiting its clinical accessibility. Manual muscle testing (MMT) is easy and quick to perform, and does not require any equipment [10], but presents poor psychometric properties [10]. Indeed, MMT lacks sensitivity to identify changes in muscle strength over time [11, 12]. Quantitative muscle testing (QMT) using a handheld dynamometer (HHD) is a promising alternative for muscle strength assessment. HHD is simple, affordable, accessible for clinicians, and more accurately detects muscle weakness than MMT [11–13]. QMT has good to excellent psychometric properties for different muscle groups evaluated in various populations [9, 14–16]. Indeed, MIMS values obtained with HHD show good concurrent validity with isokinetic dynamometry [9, 17, 18] and good to excellent reliability for most muscle groups [14, 19–24]. To be confident that muscle strength changes are true changes rather than the result of measurement error, clinicians should ensure that the measurement error of the chosen outcome measure is small [25]. This can be assessed using measurement error parameters such as the standard error of measurement (SEM), limits of agreement (LOA), and minimal detectable change (MDC) [25].

Previous studies have showed good to excellent intra- and inter-rater reliability of HHD muscle strength measurements for different numbers of muscle groups, except for the ankle muscle groups which showed moderate intra- and inter-rater reliability [14–16, 19–21, 26, 27]. However, none has assessed the intra and inter-rater reliability of a standardized HHD protocol for the assessment of muscle torque for multiple key muscle groups of the upper and lower limbs essential to achieving daily activities. Moreover, the protocols and the types of devices used in these studies have several limitations that discourage their use in research and clinical settings including overlooking the effect of gravity, not measuring the lever arm, a lack of joint stabilization especially for strong muscle groups, and a lack of device stability due to the poor ergonomics of the HHD used [28].

The objectives of this study were to determine the intra- and inter-rater reliability, agreement, SEM, and MDC of the muscle strength torque values of 17 muscle groups of the upper and lower extremities in healthy adults, obtained with a standardized protocol using a push–pull HHD. Based on the results obtained by Hébert et al. [29], our hypothesis is that intra- and inter-rater reliability will be good to excellent for all muscle groups tested.

## Methods

### Participants

A convenience sample of 30 healthy adults aged between 18 and 70 years old was used for this study. Based on data obtained in a previous intra-rater reliability study for knee extensors assessment using the same protocol (ICC = 0.98) [30] and according to the review of Bujang and Baharum [31], the sample size was determined using a 80% power, α = 0.05, minimum acceptable reliability of 0.5, and an expected good to excellent reliability > 0.75. Participants were recruited through advertisements in newspapers, social networks, contact lists of different employers, and posters placed in public areas. Participants were included if they were available to take part in the protocol spanning half a day. They were excluded if they presented any of the following criteria: 1) participation in sport at a competitive level; 2) degenerative or neuromusculoskeletal disease that could affect torque measurements; 3) traumatic experience or disease in the previous years that could affect their muscle capacity and strength; and 4) use of medication that could impact muscle strength (e.g., muscle relaxants, analgesics, opioids) at the time of the evaluation. Written informed consent was obtained from each participant prior to the first assessment, and the study was approved by the *Ethics Committee of the Integrated University Center of health and social services (CIUSSS) of the Capitale-Nationale*.

### Instrumentation

The MEDup™ HHD (Atlas Medic, Québec, Canada) was used in either compression or distraction mode depending on the muscle group evaluated. The dynamometer was set to read muscle strength values in Newtons. The calibration of the dynamometer was verified with reference weights at baseline and every 3 months to ensure validity and good measurement accuracy.

The measurements were performed by two independent raters who had received 3 full days of training on the standardized operative procedure and the HHD protocol. The training was followed by approximatively 20 h of practice. The first evaluator (E1) was a 31-year-old female physiotherapist who worked at the CIUSSS of Saguenay–Lac-St-Jean, with 4 years of clinical experience in geriatrics, and no experience using HHD. She was 5′10″ in height and weighed 63,6 kg. The second evaluator (E2) was a 23-year-old female physiotherapy technologist who worked in a private clinic, with one year of clinical experience, and no experience using HHD. She was 5’5” in height and weighed 85 kg.

### Study protocol

**Fig. 1:**
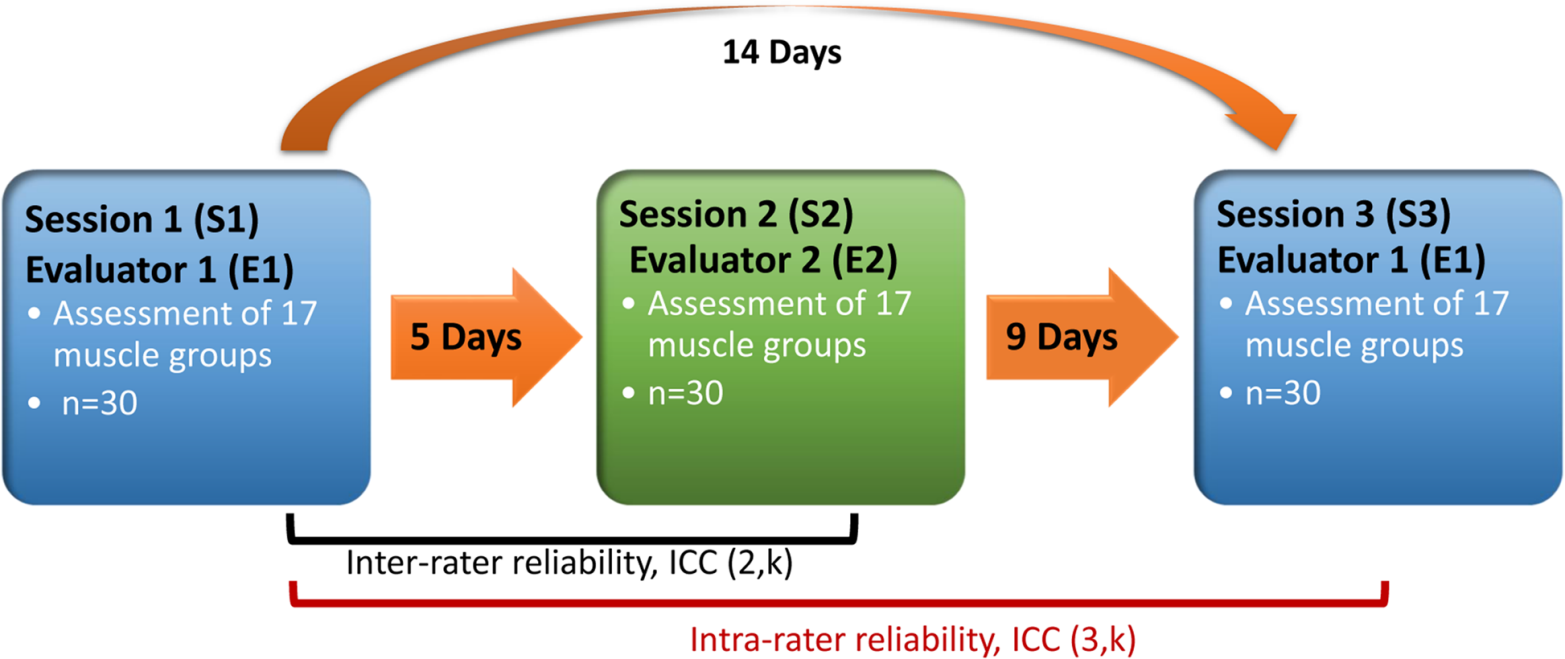
Study Protocol. Torque of 17 muscle groups was assessed by two independent raters at three different times (S1, S2, S3) over a 14-day period (*n* = 30 participants). Intra- and inter-rater reliability were determined by comparing the torque values obtained at S1 and S3 and S1 and S2 using intraclass correlation coefficients (ICC(3,k), ICC(2,k)

Data collection of this cross-sectional study was conducted from January 2021 to October 2021. MIMS torque of 17 muscle groups of the upper (shoulder abductors, internal and external rotators, and flexors; elbow and wrist flexors and extensors) and lower (hip abductors, internal and external rotators, flexors and extensors; knee flexors and extensors and ankle dorsiflexors and evertors) extremities was measured using a standardized HHD protocol inspired by a protocol previously published by Hébert et al. [29]. The current protocol is described in detail for each muscle groups (subject’s and evaluator’s position, stabilization, adapter type and dynamometer placement and lever arm measurement) in the supplementary materials (see Additional files 1, 2 and 3). As shown in Fig. 1, measurements were taken during three different sessions (S1, S2 et S3) by two independent evaluators. MIMS torque of the right or left side of the 17 muscle groups was assessed during an initial evaluation session (S1) by the first evaluator (E1). Five days later, MIMS torque of the same side was assessed in a second session (S2) by the second evaluator (E2) to assess the inter-rater reliability. Finally, nine days later, the MIMS torque of the same side was measured in a third session (S3) by the first evaluator (E1) to assess the intra-rater reliability. The order in which muscle groups were assessed for each participant was determined during the first session using bloc randomization of the upper and lower extremities and muscle groups to control for learning effect and potential fatigue. This order was subsequently reproduced for each session. The side (right or left) being evaluated was alternatively selected between consecutive participants.

### Assessment protocol

The following guiding principles were systematically applied for each muscle group tested: a. to control for the effect of gravity, each testing position was chosen to eliminate the effect of the evaluated segment's weight; b. the body of the dynamometer was aligned with the plane of movement and was perfectly perpendicular to the segment in order to register 100% of the force vector produced by the evaluated muscle group; c. to control for compensations, non-slip surfaces and rigid straps were used to stabilize and/or to perform closed chain evaluations, thus eliminating the effect of the evaluator; d. easy-to-palpate anatomical landmarks were chosen in order to accurately and reproducibly measure the lever arms, and; e. a comprehensive standardized training session for the evaluators that was long enough to allow them to integrate all these principles for each of the 17 muscle groups evaluated was provided. In each evaluation session, the limb was first placed in the testing position by the evaluator and a submaximal contraction of about 50% of the maximal effort was performed before each trial to ensure that the isometric contraction was well understood and executed, and that the stabilization of the segment was adequate. Then, the participant was asked to produce a maximal contraction by gradually pushing against the HHD (or by pulling the strap for the distraction mode), steadily increasing to their maximal effort, and maintaining the maximal effort until they were told to release. Contractions lasted for ten seconds. The following standardized verbal encouragement was given throughout the effort to ensure that the peak force was reached: “Go ahead, push, harder, push, go ahead, as hard as you can”. The intensity and tone of voice of the encouragements were gradually increased over the course of the 10-s contraction. Three trials were performed using isometric “make” tests, meaning that the evaluator holds the HHD still while the participant exerts a maximal force against it. The coefficient of variation between trials was calculated, and when it exceeded ten percent, additional trials were performed until obtaining three measures within ten percent of variation, up to a maximum of five measures. The three closest trials were kept for the final analyses. A minimum rest period of 30 s was allowed between each trial. If needed, an additional rest period was allowed to ensure that maximum strength was achieved for each trial and each muscle group. The lever arm was measured for each muscle group on each side, as described in the standard operating procedure in Additional file 1 of the supplementary material, to convert the MIMS obtained in Newtons into Newton-meter torque values. When required, rigid straps were used to: a. resist the contraction, b. inserting the HHD between the segment and the strap (hip extensors, knee extensors), c. stabilize the segment to avoid compensations (wrist flexors and extensors, hip abductors, ankle evertors), or d. perform the evaluation in distraction mode (hip flexors, hip abductors, knee flexors). Pain was assessed with a visual analogue scale, and when pain prevented the participant from reaching their maximal effort, the test was not repeated, and data were excluded from the final analysis. Evaluators make sure to correct the compensations that may occur (e.g., right body alignment, ensure that the starting position is maintained and that the stabilization is used only to stabilize and not to produce force). At the first assessment session, anthropometrical data such as age, gender, height, weight, and body mass index were also documented.

### Statistical analysis

The mean of the three torque values (obtained by multiplying the strength values [Newton] by the lever arm [meter]) of each side were calculated for all muscle groups for each participant. Descriptive statistics (mean and standard deviation [SD]) of these means were calculated. Normality of the MIMS distribution for each muscle group was analyzed using Shapiro–Wilk tests. Descriptive statistics (mean, SD, frequency, and percentage) of participant characteristics were also calculated. Intra- and inter-rater reliability were calculated using intraclass correlation coefficients (ICC) with 95% confidence intervals (CI). Intra-rater reliability was calculated by comparing measurements taken by the same rater (E1) fourteen days apart (S1 and S3), using multiple measurements in a two-way mixed-effects model with absolute agreement. Inter-rater reliability was calculated by comparing the torque values obtained by two different raters (E1 et E2) five days apart (S1 and S2), using multiple measurements in a two-way random effects model with absolute agreement. ICC were qualified according to Koo and Li (2016), proposing that ICC greater than 0.90, between 0.75 and 0.9, between 0.5 and 0.75 and less than 0.5 suggests excellent, good, moderate, and poor reliability, respectively [32]. Bland and Altman (BA) plots were also used to evaluate the agreement between the measurements taken at different sessions. One-sample t-tests of the difference of scores obtained between measurement time-points were used to identify significant systematic bias and provide all the relevant data to calculate the limits of agreement and to draw BA plots. The SEM was calculated using the following formula: SD_pooled_*√(1-ICC), where the SD_pooled_ is the average of the SD calculated from the 6 trials (3 trials in each session) for each participant [25]. MDC was also calculated with a 95% CI using the formula MDC = 1.96*SEM*√2, where 1.96 is derived from the 95% CI [25]. Pairwise deletion was applied in the presence of missing data. Significance was set at α < 0.05 and all statistical analyses were performed using SPSS (IBM SPSS Statistics 28.0 for Windows, Armonk, NY, USA).

## Results

### Participants

Fifteen women and seventeen men took part in this study. Two women dropped out after the first assessment session for personal reasons, leaving thirty participants who completed all three sessions. Participant characteristics are shown in Table 1. A minimum of 28 participants completed the three sessions for each muscle group (see Table 2). Three participants were unable to produce a maximal contraction for certain muscle groups due to pain or discomfort in specific joints (shoulder abductors and wrist flexors [*n* = 1], shoulder external rotators [*n* = 1], hip abductors and extensors [*n* = 1]). In addition, we were unable to assess a full maximal contraction of the hip flexors, internal and external rotators, and knee flexors of two participants due to a transient technical problem of the HHD in their second and third evaluation sessions. Finally, one participant's shoulder internal rotator strength could not be measured according to protocol due to the size of its abdomen.

**Table 1:** Participants’ characteristics

**Participant**	**Decade**	**Sex**	**Dominance UE**	**Dominance LE**	**Age**	**Height (cm)**	**Weight (kg)**	**BMI (kg/m**^**2**^**)**
6	20	M	R	R	29	175	63.6	20.8
19	20	M	R	R	22	178	70.5	22.2
20	20	M	R	R	28	174	86.4	28.5
76	30	M	R	R	31	194	84.1	22.3
82	30	M	R	R	31	180	95.5	29.5
113	30	F	R	R	38	172	59.1	20.0
139	30	F	R	L	37	163	57.3	21.7
149	40	M	L	R	40	175	89.1	29.1
150	40	M	R	R	42	180	65.9	20.3
190	40	F	R	R	46	172	54.1	18.3
281	60	M	R	R	66	160	56.4	22.0
289	60	M	R	R	66	176	86.4	27.9
291	60	M	R	R	65	170	67.0	23.1
292	60	M	L	L	68	180	75.0	23.1
321	60	F	R	R	62	160	59.1	23.1
186	40	F	R	R	46	165	60.5	22.2
69	20	F	L	L	27	160	56.8	22.2
324	60	F	R	R	69	168	72.6	25.8
323	60	F	R	R	60	170	65.0	22.5
187	40	F	R	R	47	170	63.6	22.0
327	60	F	R	R	67	168	62.7	22.2
70	20	F	L	L	21	173	61.4	20.6
114	30	F	R	R	30	158	61.8	24.9
64	20	F	R	R	27	162	56.8	21.8
5	20	M	L	R	24	170	59.1	20.4
16	20	M	R	R	22	185	85.0	24.8
74	30	M	R	R	37	184	85.5	25.2
23	20	M	R	R	23	180	86.0	26.5
147	40	M	R	R	40	180	81.8	25.3
24	20	M	R	R	28	178	126.4	40.0
**Mean** **(SD)**					**41.3 (16.5)**	**173** **(9)**	**71.8 (16.0)**	**24.1** **(4.1)**
**Frequency** **(%)**	20–29: *n* = 10 (33.3%)	M: *n* = 17 (57%)	L: *n* = 5 (17%)	L: *n* = 4 (13%)				
	30–39: *n* = 6 (20%)	F: *n* = 13 (43%)	R: *n* = 25 (83%)	R: *n* = 26 (87%)				
	40–49: *n* = 6 (20%)							
	60–69: *n* = 8 (26.7%)							

*M* Male, *F* Female, *R* Right, *L* Left, *UE* Upper extremity, *LE* Lower extremity, *BMI* Body Mass Index, *SD* Standard Deviation

**Table 2:** Intra- and inter-rater reliability, standard error of measurement and minimal detectable change

***Muscle Groups***	***N***	***MIMS Torque Mean (SD) in Nm***			***ICC 95%***	***CI 95%***	***P-value***	***SEM***	***MDC***
		**S1**	**S3**	**Mean (S1 + S3) / 2**	**Intra-rater reliability**			**Nm (%)**	
*Shoulder ABD*	29	80.6 (34.6)	81.1 (37.0)	80.8	0.962	0.918–0.982	< 0.001	1.30 (1.60)	3.59 (4.45)
*Shoulder IR*	29	35.4 (12.8)	35.4 (12.0)	35.4	0.988	0.974–0.994	< 0.001	0.21 (0.60)	0.59 (1.66)
*Shoulder ER*	29	26.5 (9.3)	26.8 (8.9)	26.6	0.984	0.965–0.992	< 0.001	0.20 (0.75)	0.56 (2.09)
*Shoulder flexors*	30	68.1 (25.9)	72.0 (30.46)	70.1	0.985	0.953–0.994	< 0.001	0.52 (0.75)	1.45 (2.07)
*Elbow flexors*	30	53.1 (17.9)	53.2 (16.52)	53.2	0.987	0.972–0.994	< 0.001	0.27 (0.52)	0.76 (1.43)
*Elbow extensors*	30	33.0 (12.9)	32.1 (11.9)	32.5	0.990	0.978–0.995	< 0.001	0.16 (0.50)	0.45 (1.38)
*Wrist flexors*	29	10.2 (3.1)	10.8 (3.2)	10.5	0.902	0.789–0.954	< 0.001	0.30 (2.84)	0.83 (7.88)
*Wrist extensors*	30	7.6 (2.4)	7.7 (2.7)	7.6	0.947	0.890–0.975	< 0.001	0.14 (1.80)	0.38 (4.99)
*Hip ABD*	29	125.4 (35.6)	127.8 (40.2)	126.6	0.956	0.907–0.979	< 0.001	1.90 (1.50)	5.25 (4.15)
*Hip IR*	29	72.2 (20.7)	75.4 (21.6)	73.8	0.964	0.914–0.984	< 0.001	0.90 (1.23)	2.51 (3.40)
*Hip ER*	29	54.2 (18.2)	55.4 (19.1)	54.8	0.966	0.929–0.984	< 0.001	0.70 (1.28)	1.94 (3.55)
*Hip flexors*	29	131.2 (44.5)	138.6 (47.5)	134.9	0.933	0.854–0.969	< 0.001	3.18 (2.36)	8.81 (6.53)
*Hip extensors*	29	217.6 (65.5)	221.4 (75.7)	219.5	0.961	0.917–0.981	< 0.001	3.20 (1.46)	8.87 (4.04)
*Knee flexors*	29	94.1 (33.4)	92.2 (29.7)	93.1	0.956	0.907–0.979	< 0.001	1.30 (1.39)	3.59 (3.86)
*Knee extensors*	30	149.6 (51.6)	148.4 (50.7)	149.0	0.983	0.964–0.992	< 0.001	0.96 (0.64)	2.65 (1.78)
*Ankle DF*	30	23.6 (5.6)	23.8 (5.4)	23.7	0.967	0.932–0.984	< 0.001	0.24 (1.00)	0.66 (2.78)
*Ankle evertors*	30	20.1 (6.0)	21.0 (7.1)	20.6	0.965	0.922–0.984	< 0.001	0.26 (1.27)	0.72 (3.52)
***Muscle Groups***	**N**	**S1**	**S2**	**Mean (S1 + S2) / 2**	**Inter-rater reliability**			**Nm (%)**	
*Shoulder ABD*	29	80.6 (38.5)	80.1 (35.2)	80.3	0.956	0.906–0.979	< 0.001	1.53 (1.90)	4.24 (5.28)
*Shoulder IR*	28	34.7 (12.5)	35.1 (13.2)	34.9	0.977	0.951–0.989	< 0.001	0.34 (0.98)	0.94 (2.70)
*Shoulder ER*	29	25.6 (9.03)	26.0 (8.8)	25.8	0.979	0.955–0.990	< 0.001	0.24 (0.93)	0.67 (2.58)
*Shoulder flexors*	30	68.1 (30.0)	75.4 (30.1)	71.8	0.963	0.822–0.987	< 0.001	1.13 (1.58)	3.14 (4.38)
*Elbow flexors*	30	53.1 (17.9)	54.4 (17.9)	53.8	0.988	0.974–0.994	< 0.001	0.26 (0.49)	0.73 (1.35)
*Elbow extensors*	30	33.0 (12.9)	32.6 (12.0)	32.8	0.989	0.978–0.995	< 0.001	0.17 (0.51)	0.47 (1.43)
*Wrist flexors*	29	10.2 (3.1)	9.4 (2.9)	9.8	0.888	0.731–0.950	< 0.001	0.32 (3.25)	0.88 (9.02)
*Wrist extensors*	30	7.6 (2.4)	7.5 (2.6)	7.5	0.902	0.794–0.953	< 0.001	0.23 (3.06)	0.64 (8.49)
*Hip ABD*	30	125.4 (35.0)	124.4 (35.9)	124.9	0.965	0.927–0.983	< 0.001	1.41 (1.13)	3.9 (3.12)
*Hip IR*	28	71.8 (21.0)	63.3 (20.2)	67.5	0.897	0.548–0.964	< 0.001	2.01 (2.98)	5.57 (8.25)
*Hip ER*	28	53.8 (18.3)	50.1 (18.5)	51.9	0.938	0.854–0.973	< 0.001	1.07 (2.05)	2.95 (5.69)
*Hip flexors*	28	130.5 (45.2)	123.5 (37.7)	127.0	0.964	0.908–0.985	< 0.001	1.73 (1.36)	4.80 (3.78)
*Hip extensors*	30	216.6 (64.6)	234.4 (76.8)	223.4	0.920	0.802–0.965	< 0.001	5.80 (2.57)	16.06 (7.12)
*Knee flexors*	28	93.7 (33.9)	85.5 (29)	89.6	0.931	0.805–0.972	< 0.001	1.85 (2.06)	5.13 (5.72)
*Knee extensors*	30	149.6 (51.6)	142.2 (54.8)	145.9	0.949	0.891–0.976	< 0.001	2.47 (1.69)	6.84 (4.69)
*Ankle DF*	30	23.6 (5.6)	23.7 (5.3)	23.6	0.930	0.853–0.967	< 0.001	0.40 (1.70)	1.12 (4.72)
*Ankle evertors*	30	20.1 (6.0)	18.9 (6.1)	19.5	0.944	0.859–0.975	< 0.001	0.37 (1.91)	1.03 (5.29)

Mean of the MIMS torque values with the standard deviation (SD) in Newton-meters (Nm) for each muscle group, intraclass correlation coefficients and their 95% confident intervals (ICC and CI 95%), the standard error of measurement (SEM) and the minimal detectable change (MDC) presented in Nm and in percentage (%)

*ABD* Abductors, *IR/ER* Internal and External rotators, *DF* Dorsiflexors, *S1* Session 1, *S2* Session 2, *S3* Session 3

### Intra- and inter-rater reliability

Table 2 summarizes the descriptive statistics (mean and standard deviation) of MIMS torques, intra- and inter-rater reliability, SEM, and MDC values for all muscle groups.

**Fig. 2:**
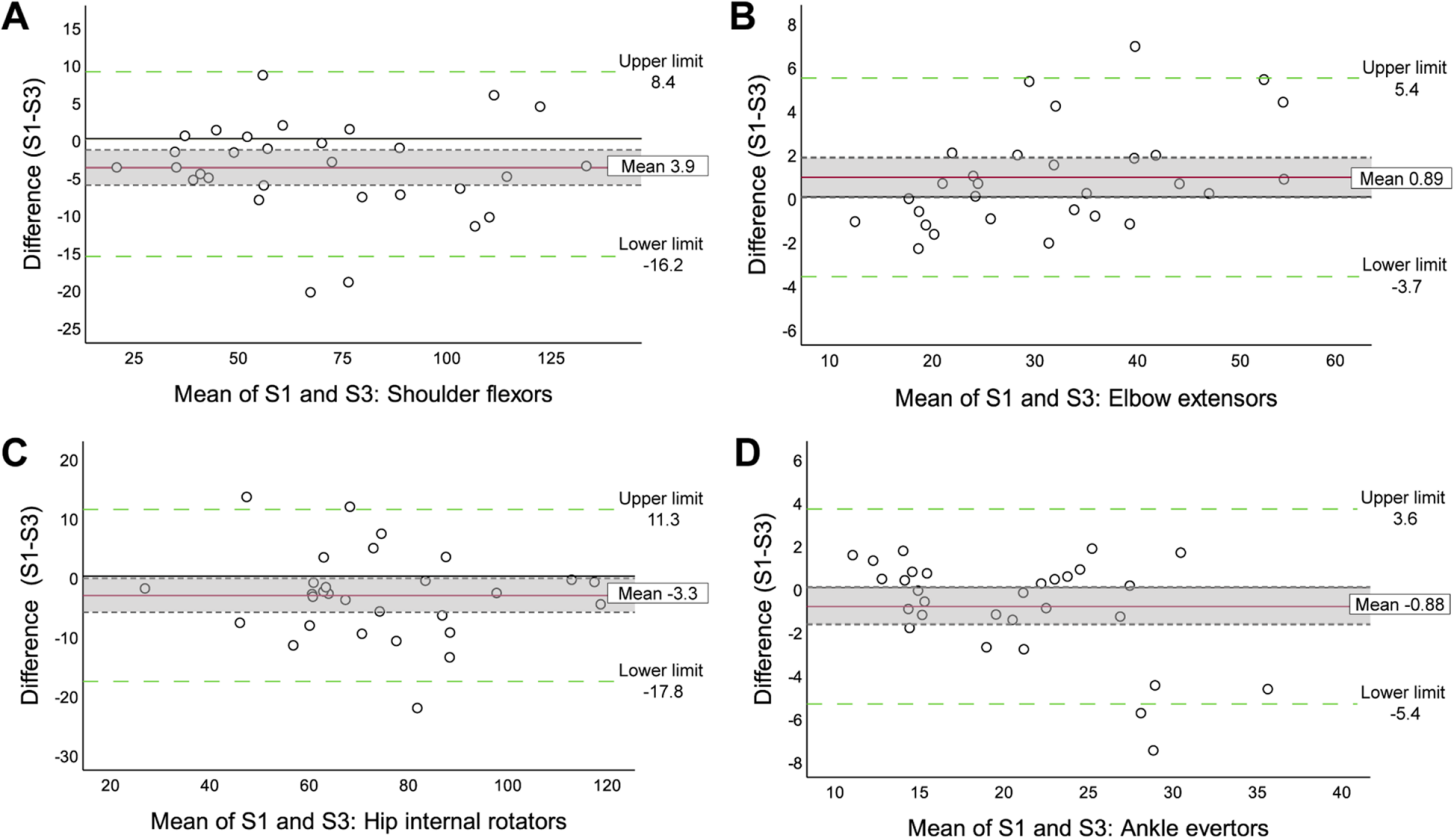
Bland and Altman plots, intra-rater assessment. Bland and Altman plots showing significant systematic bias of the mean difference of muscle torque in Nm between the first and third sessions of the shoulder flexors (**A**), elbow extensors (**B**), hip internal rotators (**C**) and ankle evertors (**D**). Limits of agreement (LOA) are identified by the dotted lines, from -1.96SD to + 1.96SD, and the mean difference by the red line. The mean difference confidence intervals are depicted by the shaded area

Regarding the intra-rater reliability, the obtained ICC values (95% CI) for all muscle groups ranged from 0.902 (0.789–0.954) to 0.990 (0.978–0.995), indicating excellent intra-rater reliability for most of the muscle groups, except for the wrist flexors and extensors and the hip flexors, which showed good to excellent reliability. Absolute and relative SEM and MDC ranged from 0.14 Nm to 3.20 Nm and 0.5% to 2.84% for the SEM, and 0.38 Nm to 8.87 Nm and 1.38% to 7.88% for the MDC, respectively, for all muscle groups (see Table 2). Table 3 shows the t-values and corresponding p-values obtained using one-sample t-tests of the differences between the measurement time-points S1-S3, and S1-S2. Only the graphs of the muscle groups that showed a systematic bias between the two-measurement time-points (S1-S3 for intra-rater reliability and S1-S2 for inter-rater reliability) are presented. Other graphs can be consulted in the supplementary material (Additional files 4 and 5). As shown in Table 3 and Fig. 2, the absolute and relative mean difference between Sessions 1 and 3 all varied from 0.01 Nm to 7.4 Nm and 0.04% to 5.6%. Only four out of 17 muscle groups (shoulder flexors, elbow extensors, internal hip rotators, ankle evertors) showed a significant systematic bias.

**Table 3:** Intra- and inter-rater agreement according to Bland and Altman plots and limits of agreement

***Muscle Groups***	***T value (Sig.)***	***Mean difference Nm (%)***	***Mean difference 95% CI in Nm***		***SD***	***Limits of agreement in Nm (95% CI)***	
			**Lower**	**Upper**		**Lower**	**Upper**
	**Intra-rater agreement (S1-S3)**						
*Shoulder ABD*	-0.2 (0.86)	-0.5 (0.6)	-6.1	5.1	14.7	-29.4 (-30.2; -28.5)	28.4 (27.5; 28.4)
*Shoulder IR*	-0.03 (0.98)	-0.01 (0.04)	-1.1	1.0	2.8	-5.5 (-5.5; -5.4)	5.4 (5.4; 5.5)
*Shoulder ER*	-0.9(0.40)	-0.4 (1.4)	-1.3	0.5	2.3	-4.9 (-5.6; -4.3)	4.2 (3.6; 4.8)
***Shoulder flexors***	**-3.4 (0.002)**	**-3.9 (5.5)**	-6.2	-1.5	**6.3**	**-16.2 (-22.9; -9.5)**	**8.4 (1.7**; **15.2)**
*Elbow flexors*	-0.1 (0.90)	-0.1 (0.2)	-1.6	1.4	4.0	-7.9 (-8.1; -7.8)	7.8 (7.6; 7.9)
***Elbow extensors***	**2,1 (0.044)**	**0.9 (2.7)**	0.0	1.8	**2.3**	**-3.7 (-5.2; -2.1)**	**5.4 (3.9; 7.0)**
*Wrist flexors*	-1.7 (0.09)	-0.6 (5.6)	-1.3	0.1	1.8	-4.1 (-5.1; -3.1)	3.0 (2.0; 4.0)
*Wrist extensors*	-0.8 (0.43)	-0.2 (2.2)	-0.6	0.3	1.1	-2.4 (-2.7; -2.1)	2.1 (1.8; 2.4)
*Hip ABD*	-0.8 (0.42)	-2.4 (1.9)	-8.4	3.6	15.7	-33.2 (-37.3; -29)	28.3 (24.2; 32.5)
***Hip IR***	**-2.4 (0.03)**	**-3.3 (4.4)**	-6.1	-0.4	**7.4**	**-17.8 (-23.4; -12.1)**	**11.3 (5.6; 16.9)**
*Hip ER*	-0.9 (0.36)	-1.2 (2.1)	-3.7	1.4	6.7	-14.4 (-16.4; -12.3)	12.0 (10.0; 14.0)
*Hip flexors*	-1.8 (0.09)	-7.4 (5.5)	-15.9	1.2	22.4	-51.3 (-64.1; -38.6)	36.6 (23.8; 49.4)
*Hip extensors*	-0.7 (0.48)	-3.7 (1.7)	-14.3	6.8	27.8	-58.2 (-64.6; -51.7)	50.7 (44.3; 57.2)
*Knee flexors*	0.8 (0.43)	1.9 (2.1)	-3.0	6.9	13.0	-23.6 (-26.9; -20.2)	27.4 (24.1; 30.8)
*Knee extensors*	0.5 (0.64)	1.2 (0.8)	-3.9	6.2	13.5	-25.2 (-27.2; -23.2)	27.5 (25.5; 29.5)
*Ankle DF*	-0.7 (0.52)	-0.2 (1.0)	-1.0	0.5	2.0	-4.1 (-4.5; -3.7)	3.6 (3.2; 3.6)
***Ankle evertors***	**-2.1 (0.046)**	**-0.9 (4.3)**	-1.7	0.0	**2.3**	**-5.4 (-6.9; -3.9)**	**3.6 (2.1; 5.2)**
***Muscle groups***	**Inter-rater agreement (S1-S2)**						
*Shoulder ABD*	-0.2 (0.86)	0.5 (0.6)	-5.4	6.4	15.4	-29.7 (-30.6; -28.9)	30.7 (29.7; 31.6)
*Shoulder IR*	-0.6 (0.57)	-0.4 (1.2)	-1.9	1.1	3.9	-8.0 (-8.7; -7.3)	7.2 (6.4; 7.9)
*Shoulder ER*	-0.8 (0.46)	-0.4 (1.4)	-1.3	0.6	2.6	-5.5 (-6.1; -4.8)	4.7 (4.1; 5.4)
***Shoulder flexors***	**-4.4(< 0.001)**	**-7.3 (10.2)**	-10.7	-3.9	**9.0**	**-24.9 (-37.6**; -12.3)	**10.4 (-2.3**; **23.0)**
*Elbow flexors*	-1.9 (0.07)	-1.3 (2.4)	-2.7	0.1	3.7	-8.6 (-10.8; -6.4)	6.0 (3.8; 8.3)
*Elbow extensors*	0.8 (0.46)	0.4 (1.1)	-0.6	1.3	2.6	-4.7 (-5.3; -4.1)	5.4 (4.8; 6.0)
***Wrist flexors***	**2.6 (0.01)**	**0.9 (8.6)**	0.2	1.5	**1.8**	**-2.6 (-4.0**; -1.1)	**4.3 (2.8**;5.7)
*Wrist extensors*	0.1 (0.93)	0.02 (0.3)	-0.5	0.6	1.5	-3.0 (-3.0; -2.9)	3.0 (3.0; 3.0)
*Hip ABD*	0.4 (0.68)	1.0 (0.8)	-3.9	5.9	13.2	-24.8 (-26.6; -23.0)	26.8 (25.1;28.6)
***Hip IR***	**4.4 (< 0.001)**	**8.5 (12.6)**	4.5	12.4	**10.2**	**-11.4 (-26.1**; 3.3)	**28.4 (13.7**; 43.1)
***Hip ER***	**2.3 (0.03)**	**3.7 (7.1)**	0.5	6.9	**8.3**	**-12.6 (-18.9**; -6.2)	**19.9 (13.6**;26.3)
***Hip flexors***	**2.6 (0.02)**	**7.0 (5.5)**	1.5	12.5	**14.2**	**-20.8 (-32.9**; -8.7)	**34.8 (22.7**; 46.8)
***Hip extensors***	**-2.8 (0.005)**	**-17.8 (8.0)**	-31.1	-4.6	**35.5**	**-87.3 (-118.2**;56.4)	**51.7 (20.8; 82.6)**
***Knee flexors***	**3.0 (0.005)**	**8.2 (9.2)**	2.7	13.8	**14.3**	**-19.8 (-34.0**; -5.6)	**36.2 (22.0**; 50.5)
*Knee extensors*	1.8 (0.09)	7.4 (5.0)	-1.1	15.9	22.8	-37.3 (-50.0; -24.5)	52.0 (39.2; 64.8)
*Ankle DF*	-0.2 (0.85)	-0.1 (0.4)	-1.2	1.0	2.8	-5.7 (-5.8; -5.5)	5.5 (5.3;5.6)
***Ankle evertors***	**2.7 (0.01)**	**1.3 (6.6)**	0.3	2.2	**2.6**	**-3.7 (-5.9**; -1.5)	**6.3 (4.1**;8.5)

T-values and corresponding *p*-values (Sig.) obtained using one-sample t-tests, the mean difference, the standard deviation (SD) and the limits of agreement (LOA) of the difference between the mean values obtained at Session 1 (S1) and Session 3 (S3) (intra-rater), and Session 1 (S1) and Session 2 (S2) (inter-rater) in Newton-meters (Nm). The mean difference of each muscle group is also expressed in percentage of the mean torque values of the two measurement time-points in parentheses. The results in bold are significant, meaning there is a significant systematic bias between the two measurement time-points

*ABD* Abductors, *IR/ER* Internal and External rotators, *DF* Dorsiflexors

**Fig. 3:**
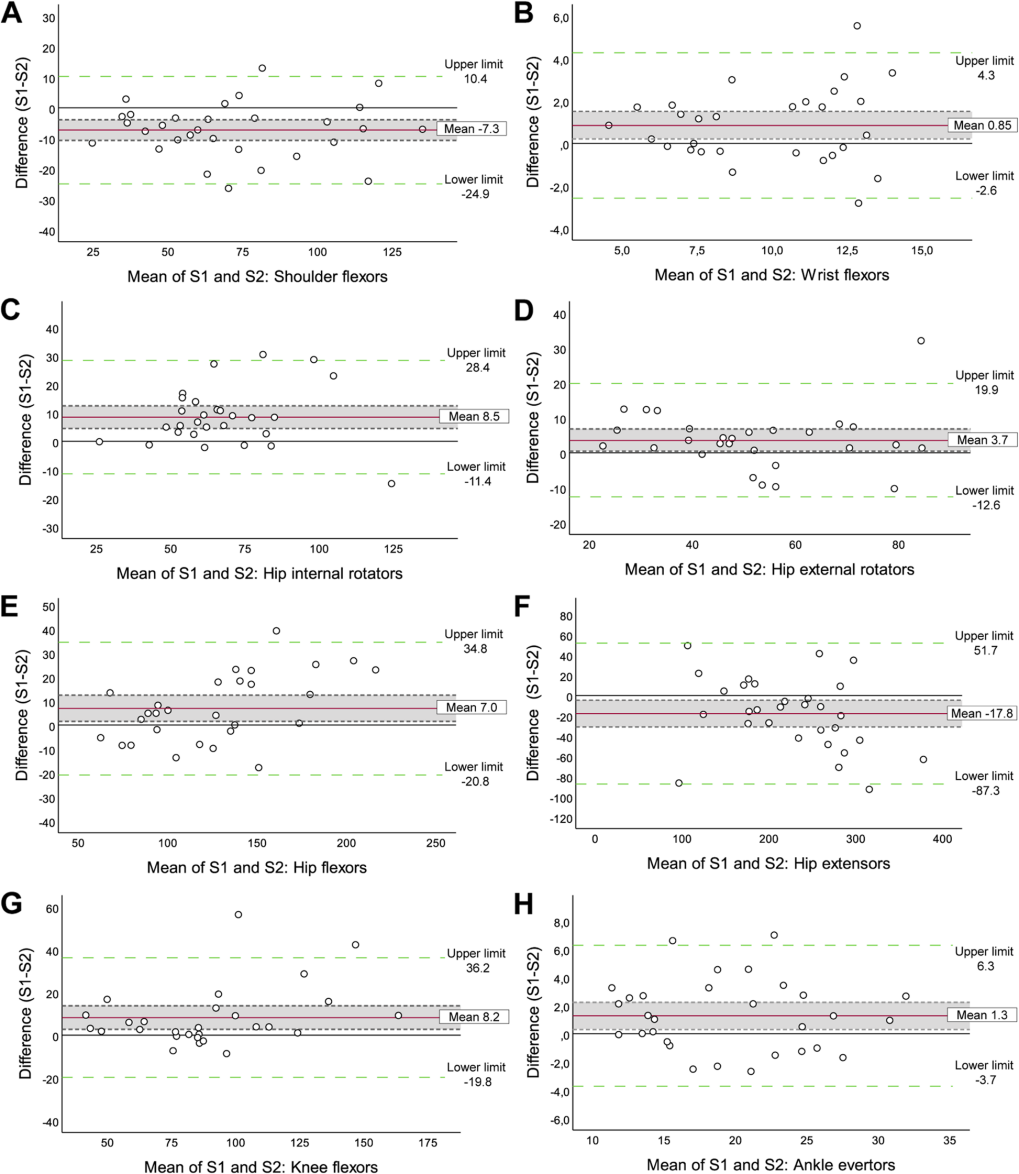
Bland and Altman plots, inter-rater assessment. Bland and Altman plots showing significant systematic bias of the mean difference of muscle torque in Nm between the first (S1) and second sessions (S2) of the shoulder flexors (**A**), wrist flexors (**B**), hip internal rotators (**C**) and external rotators (**D**), hip flexors (**E**) and extensors (**F**), knee flexors (**G**), and ankle evertors (**H**). Limits of agreement (LOA) are identified by the dotted lines, from -1.96SD to + 1.96SD and the mean difference by the full line in bold. The mean difference confidence intervals are depicted by the shaded area

Regarding the inter-rater reliability, the obtained ICC values (95% CI) ranged from 0.888 (0.731–0.950) to 0.989 (0.978–0.995) indicating good to excellent reliability for the majority (15/17) of the muscle groups tested by two different raters. Only the wrist flexors and the hip internal rotators showed moderate to excellent inter-rater reliability. Absolute and relative SEM and MDC ranged from 0.17 Nm to 5.80 Nm and 0.49% to 3.25% for the SEM, and 0.47 Nm to 16.06 Nm and 1.35% to 9.02% for the MDC, respectively. Regarding Table 3 and the BA plots (Fig. 3), the absolute values of the mean of the difference between Sessions 1 and 2 all varied from 0.02 Nm to 8.5 Nm, except for the hip extensors, which showed a mean difference of -17.8 Nm. In relative values, the mean difference for all muscle groups varied from 0.3% to 12.6% of the MIMS torque values. Eight out of 17 muscle groups showed significant systematic bias according to BA plots (see Fig. 3). Other graphs can be consulted in the supplementary material (Additional files 6 and 7).

## Discussion

The intra- and inter-rater reliability and agreement of a standardized HHD protocol for most of the muscle groups of the lower and upper limbs (*n* = 17) were documented in this study. The results demonstrate good to excellent intra- and inter-rater reliability of the protocol for almost all the muscle groups tested. To our knowledge, this is the first study to assess the intra and inter-rater reliability of a HHD protocol for such many muscle groups. Moreover, the protocol used was rigorous and respected a series of biomechanical guiding principles of muscle strength assessment that allowed us to control for many potential sources of error.

Despite our unique protocol, our results are consistent with those of certain other studies, which showed good to excellent intra- and inter-rater reliability for several muscle groups [14, 15, 19–22, 24, 26, 33–35]. However, reliability values were higher for some muscle groups, such as the ankle dorsiflexors which showed poor to good intra- and inter-rater reliability in a few other studies using HHD [14, 15, 20, 24, 26, 36]. Muscle strength assessment of the ankle dorsiflexors is challenging for a few reasons, notably: there is a short lever arm resulting in poor mechanical advantage for the evaluator, and the inclined surface of the foot in the starting position of the test makes it more difficult to position the HHD perpendicularly to the segment. The observed difference in our study could be explained in large part by the type of device used and the position of the evaluator’s wrist. Most previous studies used a MicroFET or Lafayette HHD, which are both push dynamometers and quite different from the MEDup™ used in the present study [14, 15, 20, 24, 26]. The design of the MEDup™ offers a mechanical advantage; its pistol grip (inferior handle) and bilateral handles allow a neutral wrist position and enable the evaluator to resist the participant’s force with both hands, creating better stability across muscle groups.

Concerning the wrist flexors and hip internal rotators that showed lower inter-rater ICC values, we hypothesize that more compensations (internal shoulder rotators for the wrist flexors and hip abduction for the hip internal rotators) could have occurred for these two muscle groups, potentially causing greater discrepancy between the results obtained by the two independent evaluators. Another hypothesis for the wrist flexors is that error may have been introduced using the half-sphere adaptor of the HHD, which inhibits positioning of the dynamometer support in the same place at each trial, contrary to the HHD adaptors used for all the other muscle groups. The reliability of wrist flexor HHD muscle strength assessment was only evaluated in one other study, which reported ICC values of 0.86 in healthy adults [36]. However, considering the missing data (no 95% CI provided) and the use of a different protocol in Kilmer’s study, comparisons with our results are not possible [36]. As for the reliability of the hip internal rotators, a few studies have been conducted with variable results [23, 26, 37]. Unlike our results, Gonzalez-Rosalen et al. [23] showed excellent inter-rater reliability. In contrast, Thorborg et al. [37] revealed similar results to ours, with fair to excellent inter-rater reliability and no agreement between testers. However, the measurements in these studies were taken in the prone position instead of the seated position as in our protocol, which again limits comparisons. In our experience, assessing the hip rotators in the prone position increases possible compensations in the frontal plane, such as hip abduction and adduction, and it is also more difficult to keep the leg stable at 90° of knee flexion.

The results showed small measurement errors for the 17 muscle groups, with SEM and MDC all below 4% and 10% respectively in relative values for intra- and inter-rater assessments. According to the literature, a SEM of less than 10% is clinically acceptable [38]. Although Gonzalez-Rosalen et al. [23] reported good SEM values for 15 muscle groups, their use of Newtons rather than Newton-meters prevents comparisons with other studies, including ours. Also, these SEM values do not consider the error associated with measuring the lever arm, which is key to the biomechanics of strength assessment. Few studies have used the Newton-meter as a unit of force measurement, limiting comparisons to those that have. When comparing the results obtained in relative values, our results showed smaller SEM and MDC. For example, Buckinx et al. [15] showed large measurement error with relative SEM values varying from 26.56% to 101.1% for intra-observer and 17.11% to 115.29% for inter-observer. Mentiplay et al. [16], who evaluated intra- and inter-rater reliability of HHD for the assessment of isometric lower limb muscle strength found SEM varying from 5.29% to 10.81% and 4.54% to 12.53%, respectively. Altogether, studies that calculated MDC reported values greater than 10% for all muscle groups tested [15, 19, 39, 40] even if they only measured muscle strength values rather than torque values. By adding the lever arm measurement, one could expect the MDCs to be even higher considering that it adds another source of measurement error. These results highlight the excellent psychometric properties of our standardized HHD protocol.

Lastly, intra and inter-rater agreements using BA plots were determined to improve clinical interpretation of the agreement between the sets of measures and to validate the level of agreement quantified by the ICC [41]. Despite the high ICC values obtained for all muscle groups, no agreement between the measurements of four and eight muscle groups in intra- and inter-rater assessment, respectively, were found, which shows systematic biases between sessions and/or between testers. For the inter-rater assessment, a positive significant bias between testers was observed for a few specific muscle groups (wrist flexors, hip internal and external rotators and flexors, knee flexors, ankle evertors), meaning that E1 overestimated values compared to E2. The opposite was observed for shoulder flexors and hip extensors. Among the factors that could cause these biases, anthropometric characteristics and physical capacities of the raters could explain the perceived difference for certain muscle groups requiring greater ability to resist due to their greater strength, such as the shoulder flexors and the hip and knee flexors. Indeed, Gonzalez-Rosalen et al. [23], who compared pull and push dynamometry, found that pull dynamometry had better agreement between testers than push dynamometry, especially for stronger muscle groups due to the reduction of the examiner’s strength interaction in pull dynamometry. Also, some studies revealed significant systematic biases between raters that could be due to their capacity to resist stronger muscle groups [26, 27, 37]. However, in contrast to these studies, it is impossible to affirm that one evaluator rated systematically lower than the other. An analysis of our BA plots shows an increase in the magnitude of the mean difference with increasing mean torque values more specifically for the wrist, hip and knee flexors in inter-rater assessment, as seen in Fig. 3. This increase could be related to the smaller rater’s ability to resist greater levels of strength. Nevertheless, evaluator characteristics alone cannot explain all the differences. For some muscle groups, the role of the evaluator is less important and even zero (when assessed in a closed chain like for the knee extensors) and the assessment quality mainly relies on the positioning and stabilization of the HHD, as for the hip internal rotators and the hip extensors. Yet, these muscle groups show the greatest bias. Many other factors may come into play, such as positioning, participant compensations, and verbal stimulation. However, the standardized operating procedure should minimize such variability. These results demonstrate that this HHD protocol could still benefit from revisions to improve agreement between data, but the results obtained are much better than those of other studies [14, 15, 24, 26, 36]. This can be explained by the rigorous and novel approach of this study's protocol which is based on basic biomechanical concepts that do not seem to have been mentioned in the literature to date. The strict adherence to these guiding principles helps to control for errors associated with the handling of the HHD during testing and the data collection procedure. Consequently, the assessment of muscle strength with HHD allows reliable measurements even with inexperienced evaluators who have been appropriately trained.

This study present limitations. Although criterion validity of this standard operating procedure has been assessed in a pediatric population, it has not yet been assessed in the adult population. It would have been appropriate to do this in conjunction with the assessment of intra- and inter-rater reliability, but this would have required many additional resources and it was not the primary objective of our study. However, this step could be done in a future research project. The study sample size prevented analysis of the results by age categories and by sex. Such analysis would have facilitated use of the reference values established from our protocol. Since the measurements were taken in healthy adults with a well-defined procedure, the findings of this study cannot be generalized to other populations or types of protocols using different devices and/or different positioning.

## Conclusion

Considering the excellent intra- and inter-rater reliability and the small error of measurement of the standardized HHD protocol for 17 muscle groups, the HHD protocol is a method of choice for MIMS torque measurements in clinical and research settings. Knowing the psychometric properties of MIMS torque values obtained with this HHD standardized measurement protocol will allow optimal use of the upcoming reference values.

## Supplementary Information

**Additional file 1.** Description of the standardized HHD protocol.

**Additional file 2. **Upper limbs assessment. Legend: Muscle torque assessment of the shoulder abductors (A), shoulder internal rotators (B), shoulder external rotators (C), shoulder flexors (D), elbow flexors (E), elbow extensors (F), wrist flexors (G) and wrist extensors (H), using the MEDup^TM^.

**Additional file 3. **Lower limbs assessment. Legend: Muscle torque assessment of the hip abductors (A), hip internal rotators (B), hip external rotators (C), hip flexors (D), hip extensors (E), knee flexors (F), knee extensors(G), ankle dorsiflexors(H), and ankle evertors (I), using the MEDup^TM^.

**Additional file 4. **Bland and Altman plots, intra-rater assessment, upper limbs. Legend: Bland and Altman plots showing significant systematic bias of the mean difference of muscle torque in Nm between the first (S1) and third sessions (S3) of the shoulder abductors (A), shoulder internal and external rotators (B-C), elbow flexors (D), wrist flexors (E) and extensors (F). Limits of agreement (LOA) are identified by the dotted lines, from -1.96SD to +1.96SD and the mean difference by the full line in bold. The mean difference confidence intervals are depicted by the shaded area.

**Additional file 5. **Bland and Altman plots, intra-rater assessment, lower limbs. Legend: Bland and Altman plots showing significant systematic bias of the mean difference of muscle torque in Nm between the first (S1) and third sessions (S3) of the hip abductors (A), hip external rotators (B), hip flexors (C), hip extensors (D), knee flexors (E) and extensors (F), and ankle dorsiflexors (G). Limits of agreement (LOA) are identified by the dotted lines, from -1.96SD to +1.96SD and the mean difference by the full line in bold. The mean difference confidence intervals are depicted by the shaded area.

**Additional file 6. **Bland and Altman plots, inter-rater assessment, upper limbs. Legend: Bland and Altman plots showing significant systematic bias of the mean difference of muscle torque in Nm between the first (S1) and second sessions (S2) of the shoulder abductors (A), shoulder internal rotators(B), elbow flexors (C), shoulder external rotators (D), elbow extensors (E), and wrist extensors (F). Limits of agreement (LOA) are identified by the dotted lines, from -1.96SD to +1.96SD and the mean difference by the full line in bold. The mean difference confidence intervals are depicted by the shaded area.

**Additional file 7. **Bland and Altman plots, inter-rater assessment, lower limbs. Legend: Bland and Altman plots showing significant systematic bias of the mean difference of muscle torque in Nm between the first (S1) and second sessions (S2) of the hip abductors (A), knee extensors (B), and ankle dorsiflexors (C). Limits of agreement (LOA) are identified by the dotted lines, from -1.96SD to +1.96SD and the mean difference by the full line in bold. The mean difference confidence intervals are depicted by the shaded area.

## Data Availability

The datasets used and/or analysed during the current study could be made available from the corresponding author depending on the nature of the request.

## Abbreviations

BA: Bland and Altman
CI: Confidence interval
CIUSSS: *Centre intégré* *universitaire* *de santé et de services sociaux*
E1: Evaluator 1
E2: Evaluator 2
HHD: Hand-held dynamometry
ICC: Intra-class correlation coefficient
LOA: Limits of agreement
MDC: Minimal detectable change
MIMS: Maximal isometric muscle strength
MMT: Manual muscle testing
Nm: Newton-meter
QMT: Quantitative Muscle testing
SD: Standard deviation
SEM: Standard error of measurement
S1: Session 1
S2: Session 2
S3: Session 3
