# Supplementary material for: Psychometric properties of a standardized protocol of muscle strength assessment by hand-held dynamometry in healthy adults: a reliability study
Source: BMC Musculoskelet Disord. 2023 Apr 14;24:294. doi: 10.1186/s12891-023-06400-2 (PMC10103411; doi:10.1186/s12891-023-06400-2)
Supplement: Supplementary file 1 — Additional file 1. Description of the standardized HHD protocol. [file 12891_2023_6400_MOESM1_ESM.pdf]

Additional file 1: Description of the standardized HHD protocol

Muscle groups	Subject's position	Evaluator's position	Stabilization	Adapter type and dynamometer placement	Lever arm
Shoulder abduction	Supine<br><br>Hips and knees flexed with feet flat on the table<br><br>Arm on the tested side alongside the body<br><br>Elbow at 0° extension<br><br>Towel placed under the elbow and forearm to avoid shoulder extension	Standing in a forward lunge on the tested side<br><br>Holding the HHD with one hand on the pistol grip (inferior handle) and the other hand on the lateral handle<br><br>Pressing the pistol grip firmly against the edge of the table	Subject holds the edge of the table with the opposite hand.	Curve adapter<br><br>Just proximal to the lateral epicondyle of the elbow or just proximal to the styloid process of the ulna for stronger subjects	Distance between the postero-lateral edge of the acromion and the distal edge of the HHD adapter<br><br>Subtract half the width of the adapter surface
Shoulder internal rotators	Supine, close to the edge of the table<br><br>Hips and knees flexed and feet flat on the table<br><br>Arm on the tested side alongside the body<br><br>Elbow flexed to 90° and forearm in neutral pro/supination<br><br>Shoulder in neutral rotation<br><br>Towel placed under the elbow to avoid shoulder extension	Standing in a forward lunge on the non-tested side<br><br>Holding the HHD with one hand on the pistol grip (inferior handle) and the other hand on the lateral handle<br>or<br>both hands holding the body of the HHD	Subject holds the edge of the table with the opposite hand	Curve adapter<br><br>Palmar side of the forearm, just proximal to the styloid process of the ulna	Distance between the lateral epicondyle of the elbow and the distal edge of the HHD adapter<br><br>Subtract half the width of the adapter surface
Shoulder external rotators	Supine<br><br>Hips and knees flexed and	Standing in a forward lunge on the tested side.	Subject holds the edge of the table with the opposite hand	Curve adapter<br><br>Dorsal side of the forearm,	Distance between the lateral epicondyle of the elbow and the distal

	feet flat on the table<br><br>Arm on the tested side alongside the body<br><br>Elbow flexed to 90° and forearm in neutral pro/supination<br><br>Shoulder in neutral rotation<br><br>Towel placed under the elbow to avoid shoulder extension	Holding the HHD with one hand on the pistol grip (inferior handle) and the other hand on the lateral handle		just proximal to the styloid process of the ulna	edge of the HHD adapter<br><br>Subtract half the width of the adapter surface
Shoulder flexors	Sitting on the table with the tested side close to the edge, legs stretched out, back supported and almost vertical<br><br>Pillow under the knees<br><br>Arm alongside the body, hanging off the table<br><br>Shoulder to 0° of flexion and neutral rotation<br><br>Elbow in full extension and forearm in neutral pro/supination	Standing in a forward lunge on the tested side<br><br>Facing the tested limb to resist shoulder flexion<br><br>Holding the HHD with the hand closest to the table on the pistol grip (inferior handle) and the other hand on the lateral handle	Subject holds the table with the opposite hand	Curve adapter<br><br>Proximal to the joint line of the elbow	Distance between the postero-lateral edge of the acromion and the proximal edge of the HHD adapter<br><br>Add half the width of the adapter surface
Elbow flexors	Supine and oriented obliquely on the table so that the palmar side of the forearm (in supination) is facing slightly out when the elbow is flexed	Standing in a forward lunge on the tested side<br><br>Facing the palmar side of the forearm to resist elbow flexion	Subject holds the table with the opposite hand	Curve adapter<br><br>Palmar side of the forearm, proximal to the styloid process of the ulna	Distance between the lateral epicondyle of the elbow and the distal edge of the HHD adapter

	Hips and knees flexed and feet flat on the table<br><br>Arm on the tested side alongside the body<br><br>Elbow flexed to 90° and forearm in full supination<br><br>Shoulder in neutral rotation<br><br>Towel placed under the elbow to avoid shoulder extension	Holding HHD with the hand closest to the table on the pistol grip (inferior handle) and the other hand on the lateral handle			Subtract half the width of the adapter surface
Elbow extensors	Supine and oriented obliquely on the table so that the ulnar side of the forearm is facing slightly out when the elbow is flexed<br><br>Hips and knees flexed and feet flat on the table<br><br>Arm on the tested side alongside the body<br><br>Elbow flexed to 90° and forearm in neutral pro/supination<br><br>Shoulder in neutral rotation<br><br>Towel placed under the elbow to avoid shoulder extension	Standing in a forward lunge on the tested side<br><br>Facing the dorsal side of the forearm to resist elbow extension<br><br>Holding HHD with the hand closest to the table on the pistol grip (inferior handle) and the other hand on the lateral handle	Subject holds the table with the opposite hand	Curve adapter<br><br>Ulnar side of the forearm, proximal to the styloid process of the ulna	Distance between the lateral epicondyle of the elbow and the distal edge of the HHD adapter<br><br>Subtract half the width of the adapter surface of the HHD

Wrist flexors	Sitting close to the lateral edge of the table. The height of the table is adjusted to ensure comfort of the subject and the evaluator<br><br>Elbow and forearm supported on the table and wrist off the table<br><br>The limb is stabilized with a strap around the dorsal side of the distal forearm<br><br>Elbow flexed between 45° and 90°<br><br>Forearm and wrist in neutral position<br><br>The hand on the tested side is off of the table, relaxed in neutral flexion/extension	Sitting, facing the subject's palm<br><br>Forearm and elbow supported on the thigh on the side holding the inferior handle of the HHD<br><br>Holding the HHD with the hand closest to the table on the pistol grip (inferior handle) and the other hand on the lateral handle.<br><br>The lateral handle on the subject's side rests on the table for more stability.	The stabilization strap around the forearm is secured under the table to avoid lateral displacement of the limb when the evaluator is resisting wrist flexion<br><br>Subject stabilizes the tested forearm on the table with the opposite hand to avoid compensation in internal rotation of the shoulder when attempting wrist flexion	Half sphere adapter<br><br>Center of the palm	Distance between the joint line of the wrist and the center of the palm
Wrist extensors	Sitting close to the lateral edge of the table. The height of the table is adjusted to ensure comfort of the subject and the evaluator<br><br>Elbow and forearm supported on the table and wrist off the table<br><br>The limb is stabilized with a	Sitting, facing the dorsal side of the subject's hand<br><br>Forearm and elbow supported on the thigh on the side of the inferior handle of the HHD<br><br>Holding HHD with the hand closest to the table on the pistol grip (inferior handle) and the other hand on the	The stabilization strap around the forearm is secured under the table to avoid medial displacement of the limb when the evaluator is resisting wrist extension<br><br>Subject stabilizes the tested forearm on the table with the opposite	Large flat adapter<br><br>Metacarpal heads	Distance between the joint line of the wrist and the distal edge of the HHD adapter<br><br>Subtract half the width of the adapter surface

	strap around the palmar side of the distal forearm<br><br>Elbow flexed between 45° and 90°<br><br>Forearm and wrist in neutral position<br><br>Hand is off of the table and open with fingers in neutral flexion/extension	lateral handle.<br><br>The lateral handle on the subject's side rests on the table for more stability.	hand		
Hip abduction	Supine, close to the edge of the table on the tested side with legs extended<br><br>Arms alongside the body	Standing on the opposite side of the tested limb<br><br>The pistol grip (inferior handle) is held against the edge of the table<br><br>The HHD is positioned so that the hook adapter is aligned with the center of the non-tested thigh	The non-tested thigh is stabilized with a strap secured under the table to avoid hip abduction<br><br>The subject holds the edge of the table with both hands	Hook adapter<br><br>The hook adapter is attached to the tested limb with a second strap encircling both thighs just above the patella<br><br>Test is performed in traction mode	Distance between the superior edge of the greater trochanter and the distal edge of the strap<br><br>Subtract half the width of the strap
Hip internal rotators	Sitting at the end of the table<br><br>Knee at 90° of flexion<br><br>The back of the tested leg should not touch the edge of the table	Sitting, facing the lateral side of the tested leg<br><br>Holding the HHD in place with both hands. The lateral handles are held against the evaluator's legs, below the tibial tuberosities, for more stability. The table height is adjusted accordingly.	The subject stabilizes the tested limb by holding the edge of the table, with the forearms held on either side of the thigh to avoid hip abduction/adduction	Curve adapter<br><br>Distal to the mark on the skin made 10 cm above the tip of the lateral malleolus.	Distance between the superior edge of the lateral plateau of the tibia and the proximal edge of the HHD adapter<br><br>Add half the width of the adapter surface of the HHD
Hip external rotators	Sitting at the end of the table<br><br>Lower limb on the non-	Sitting, facing the medial side of the tested limb.<br><br>Holding the HHD in place	The subject stabilizes the tested limb by holding the edge of the table, with the	Curve adapter<br><br>Distal to the mark on the skin made 10 cm above the	Distance between the superior edge of the lateral plateau of the tibia and the proximal

	tested side in full abduction to allow the evaluator to access the medial side of the leg<br><br>Knee at 90° of flexion<br><br>The back of the tested leg should not touch the edge of the table	with both hands. The lateral handles are held against the evaluator's legs, below the tibial tuberosities, for more stability. The table height is adjusted accordingly.	forearms held on either side of the thigh to avoid hip abduction/adduction.	tip of the lateral malleolus	edge of the HHD adapter<br><br>Add half the width of the adapter surface of the HHD
Hip flexors	Standing at the end of the table, leaning forward with the antero-superior iliac spine supported on the edge of the table<br><br>Forearms supported on the table, elbows at 90° of flexion and holding the edge of the table with both hands<br><br>The hip on the tested side is flexed and the thigh is vertical<br><br>The forefoot on the tested side is in contact with the floor (toe touch)<br><br>The height of the table is adjusted so that the knee of the tested limb is flexed to about 45°<br><br>The foot of the non-tested limb is in full contact with	Sitting behind the subject<br><br>Holding the HHD with one hand<br><br>The lateral handles of the HHD are held behind the evaluator's knees at the height of the popliteal fossae<br><br>The evaluator can hold the table with one hand for stability when testing stronger subjects	The subject holds the edge of the table with both hands for stability<br><br>Non-slip membranes are placed under the subject's non-tested foot and the evaluator’s feet to avoid slipping during testing	Hook adapter<br><br>The hook adapter is attached to the tested limb with a strap around the thigh placed just above the patella<br><br>Test is performed in traction mode	Distance between the superior edge of the greater trochanter and the proximal edge of the strap<br><br>Add half the width of the strap

	the floor				
Hip extensors	Standing at the end of the table, leaning forward with the antero-superior iliac spine supported on the edge of the table<br><br>Forearms supported on the table, elbows at 90° of flexion and holding the edge of the table with both hands<br><br>The hip on the tested side is flexed and the thigh is vertical<br><br>The foot on the tested side is in full contact with the floor<br><br>The height of the table is adjusted so that the knee of the tested limb is close to 0° of flexion<br><br>The foot of the non-tested limb is in full contact with the floor	Sitting on a small bench, kneeling or crouching behind the subject<br><br>Holding the HHD with one hand<br><br>The other hand is held very close to the calf of the tested limb (but not in contact) for safety purposes<br><br>* Note that for this test, all handles of the HHD are removed	The subject holds the table with both hands for stability<br><br>A non-slip membrane is placed under the subject's non-tested foot to avoid slipping during testing	Curve adapter<br><br>Distal to the mark on the skin made 10 cm above the tip of the lateral malleolus<br><br>The HHD is inserted between the evaluated limb and a strap which is secured under the table	Distance between the superior edge of the greater trochanter and the proximal edge of the strap<br><br>Add half the width of the strap
Knee flexors	Sitting at the end of the table<br><br>Knee at 90° of flexion<br><br>The back of the leg should not touch the edge of the	Sitting in front of the subject<br><br>The lateral handles of the HHD are held behind the evaluator's knees at the height of the popliteal fossae	The subject holds the edge of the table on either side of the tested limb<br><br>A non-slip membrane is placed under the evaluator's feet to	Hook adapter<br><br>Distal to the mark on the skin made 10 cm above the tip of the lateral malleolus<br><br>The hook adapter is attached to the tested limb	Distance between the superior edge of the lateral tibial plateau and the proximal edge of the strap<br><br>Add half the width of the strap

	table	The evaluator can hold the table with one hand for stability when testing stronger subjects	avoid slipping during testing	with a strap around the leg placed just below the mark on the skin<br><br>Test is performed in traction mode	
Knee extensors	Sitting at the end of the table<br><br>Knee at 90° of flexion<br><br>The back of the leg should not touch the edge of the table	Sitting on a small bench, kneeling or crouching behind the subject<br><br>Holding the HHD with one hand<br><br>The other hand is held very close to the lower leg of the tested limb (but not in contact) for safety purposes<br><br>* Note that for this test, all handles of the HHD are removed	The subject leans slightly backward and holds the edges of the table for stability and to avoid lifting the buttocks	Curve adapter<br><br>Distal to the mark on the skin made 10 cm above the tip of the lateral malleolus<br><br>The HHD is inserted between the evaluated limb and a strap which is secured under the table	Distance between the superior edge of the lateral tibial plateau and the proximal edge of the HHD adapter<br><br>Add half the width of the adapter surface of the HHD
Ankle dorsiflexors	Supine, close to the edge of the table on the tested side with legs extended<br><br>A pillow or rolled towel is inserted under the knees<br><br>Feet off of the table<br><br>Ankle at 0° of dorsiflexion (neutral between dorsiflexion and plantar flexion)	Standing in a forward lunge on the tested side<br><br>Holding the HHD with the hand closest to the table on the pistol grip (inferior handle) and the other hand on the lateral handle<br><br>The arm holding the pistol grip can rest on the subject's leg	The subject holds the edges of the table with both hands for stability	Large flat adapter<br><br>Dorsal side of the foot over the metatarsal heads, perpendicular to the foot's axis	Perpendicular distance between a line extending the long axis of the fibula and a mark corresponding to the distal edge of the HHD adapter<br><br>Subtract half the width of the adapter surface of the HHD
Ankle evertors	Supine with the leg on the tested side extended and the hip and knee of the non-	Sitting on the tested side facing the lateral side of the ankle	The tested leg is stabilized with a strap placed around the	Curve adapter<br><br>Proximal to the distal edge	Perpendicular distance between a line extending the long axis

	tested side flexed with foot flat on the table<br><br>A pillow or rolled towel is inserted under the knee on the tested side<br><br>The foot on the tested side is off of the table and the ankle is at 0° of dorsiflexion (neutral between dorsiflexion and plantar flexion)	Holding the HHD with one hand on the pistol grip (inferior handle) and the other hand on the lateral handle	subject's leg (just above the ankle) and around the evaluator's waist to avoid medial displacement of the limb when the evaluator is resisting ankle eversion	of the 5th metatarsal head	of the fibula and a mark corresponding to the distal edge of the HHD adapter<br><br>Subtract half the width of the adapter surface of the HHD
