# Supplementary figures and images for: Psychometric properties of a standardized protocol of muscle strength assessment by hand-held dynamometry in healthy adults: a reliability study
Source: BMC Musculoskelet Disord. 2023 Apr 14;24:294. doi: 10.1186/s12891-023-06400-2 (PMC10103411; doi:10.1186/s12891-023-06400-2)

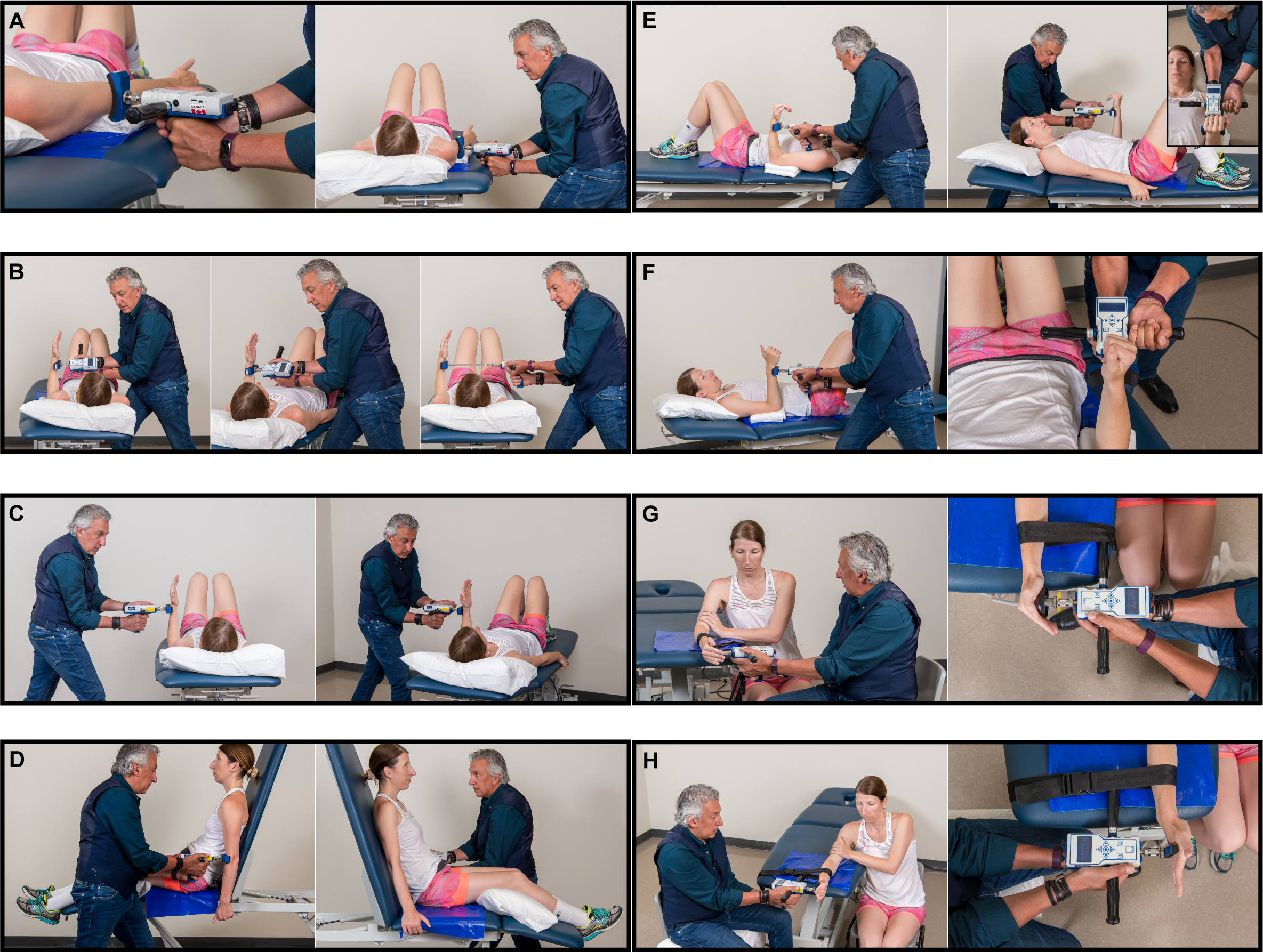

Supplement: Supplementary file 2 — Additional file 2. Upper limbs assessment. Legend: Muscle torque assessment of the shoulder abductors (A), shoulder internal rotators (B), shoulder external rotators (C), shoulder flexors (D), elbow flexors (E), elbow extensors (F), wrist flexors (G) and wrist extensors (H), using the MEDupTM. [file 12891_2023_6400_MOESM2_ESM.tif]

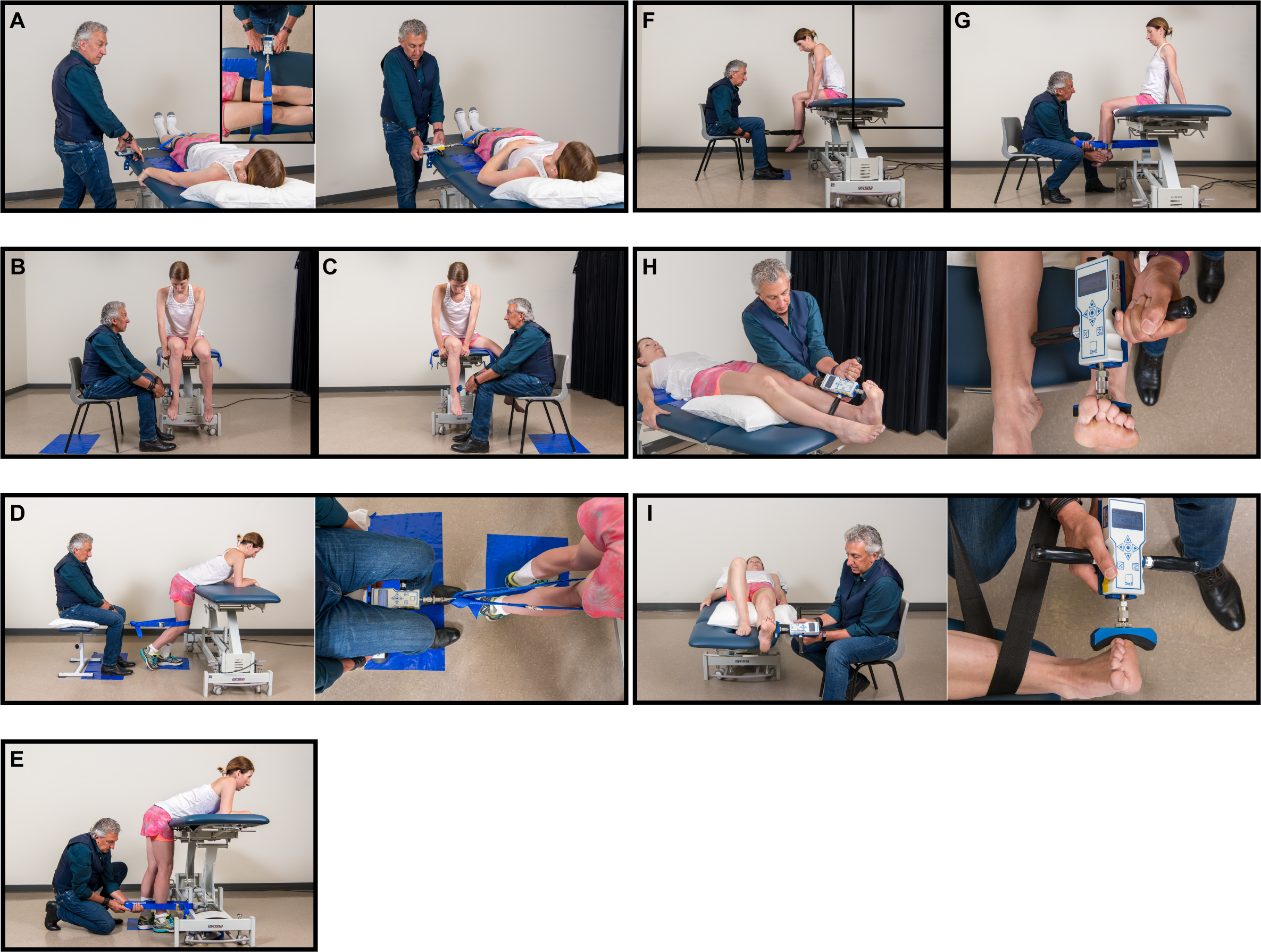

Supplement: Supplementary file 3 — Additional file 3. Lower limbs assessment. Legend: Muscle torque assessment of the hip abductors (A), hip internal rotators (B), hip external rotators (C), hip flexors (D), hip extensors (E), knee flexors (F), knee extensors(G), ankle dorsiflexors(H), and ankle evertors (I), using the MEDupTM. [file 12891_2023_6400_MOESM3_ESM.tif]

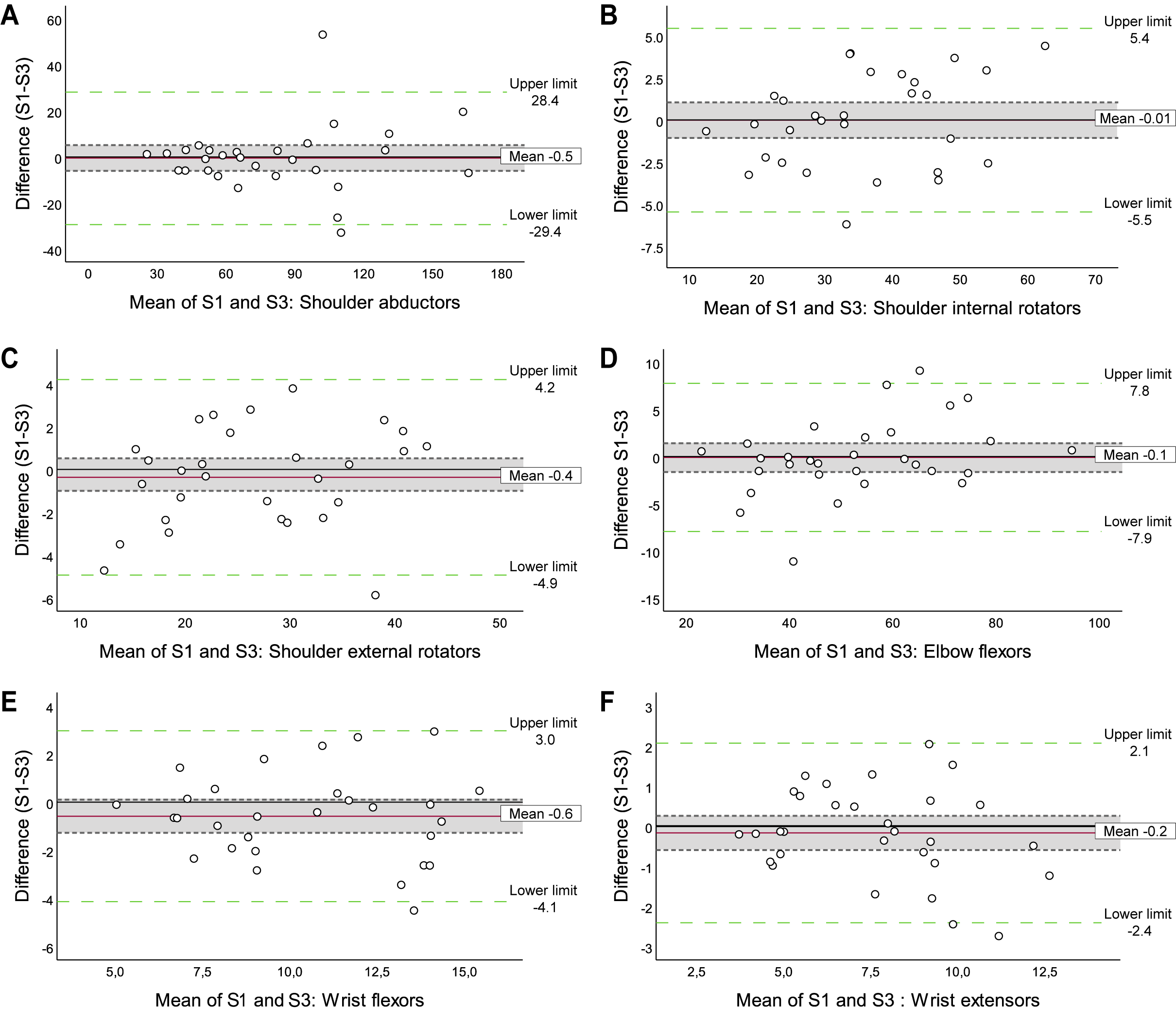

Supplement: Supplementary file 4 — Additional file 4. Bland and Altman plots, intra-rater assessment, upper limbs. Legend: Bland and Altman plots showing significant systematic bias of the mean difference of muscle torque in Nm between the first (S1) and third sessions (S3) of the shoulder abductors (A), shoulder internal and external rotators (B-C), elbow flexors (D), wrist flexors (E) and extensors (F). Limits of agreement (LOA) are identified by the dotted lines, from -1.96SD to +1.96SD and the mean difference by the full line in bold. The mean difference confidence intervals are depicted by the shaded area. [file 12891_2023_6400_MOESM4_ESM.tif]

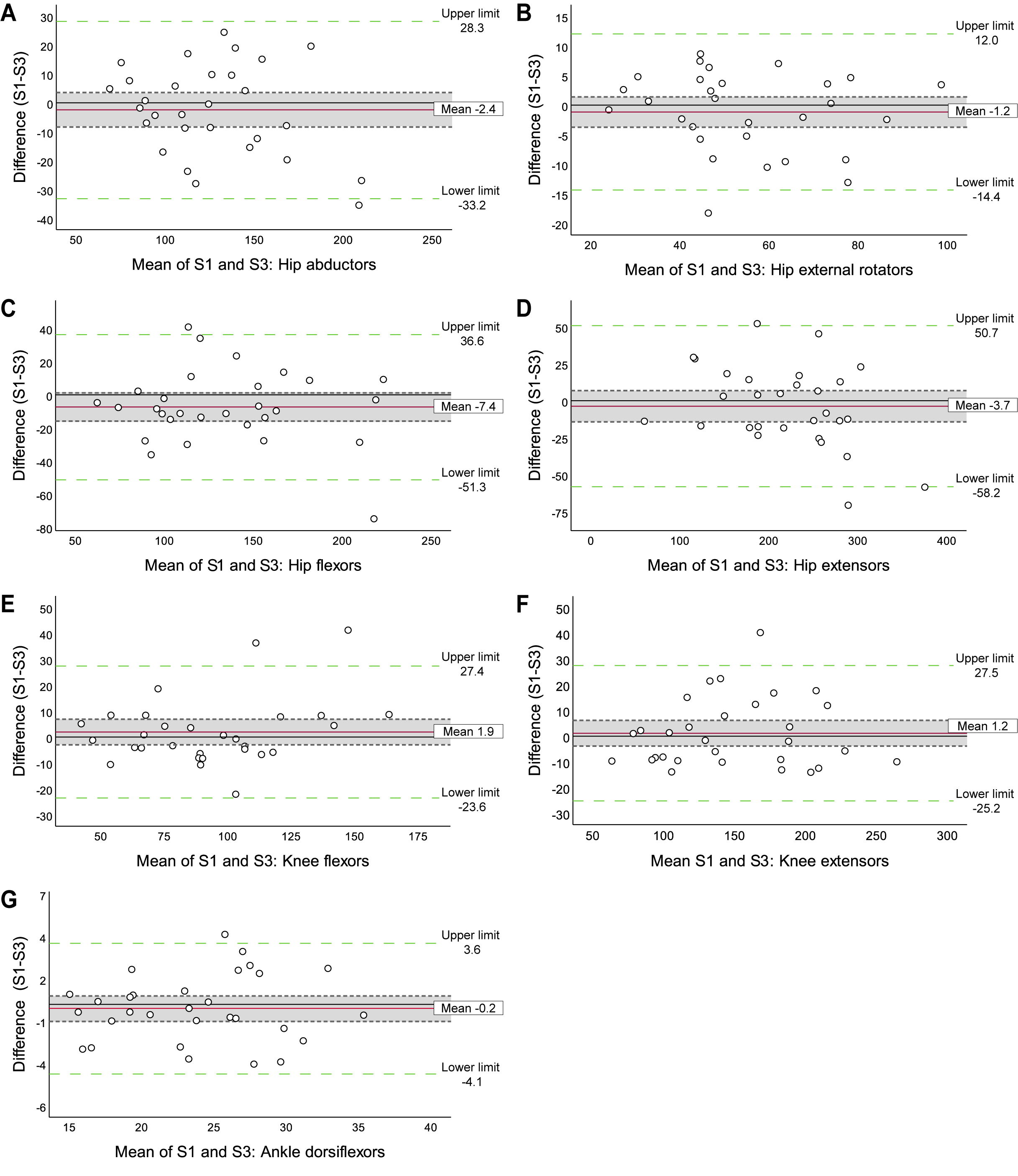

Supplement: Supplementary file 5 — Additional file 5. Bland and Altman plots, intra-rater assessment, lower limbs. Legend: Bland and Altman plots showing significant systematic bias of the mean difference of muscle torque in Nm between the first (S1) and third sessions (S3) of the hip abductors (A), hip external rotators (B), hip flexors (C), hip extensors (D), knee flexors (E) and extensors (F), and ankle dorsiflexors (G). Limits of agreement (LOA) are identified by the dotted lines, from -1.96SD to +1.96SD and the mean difference by the full line in bold. The mean difference confidence intervals are depicted by the shaded area. [file 12891_2023_6400_MOESM5_ESM.tif]

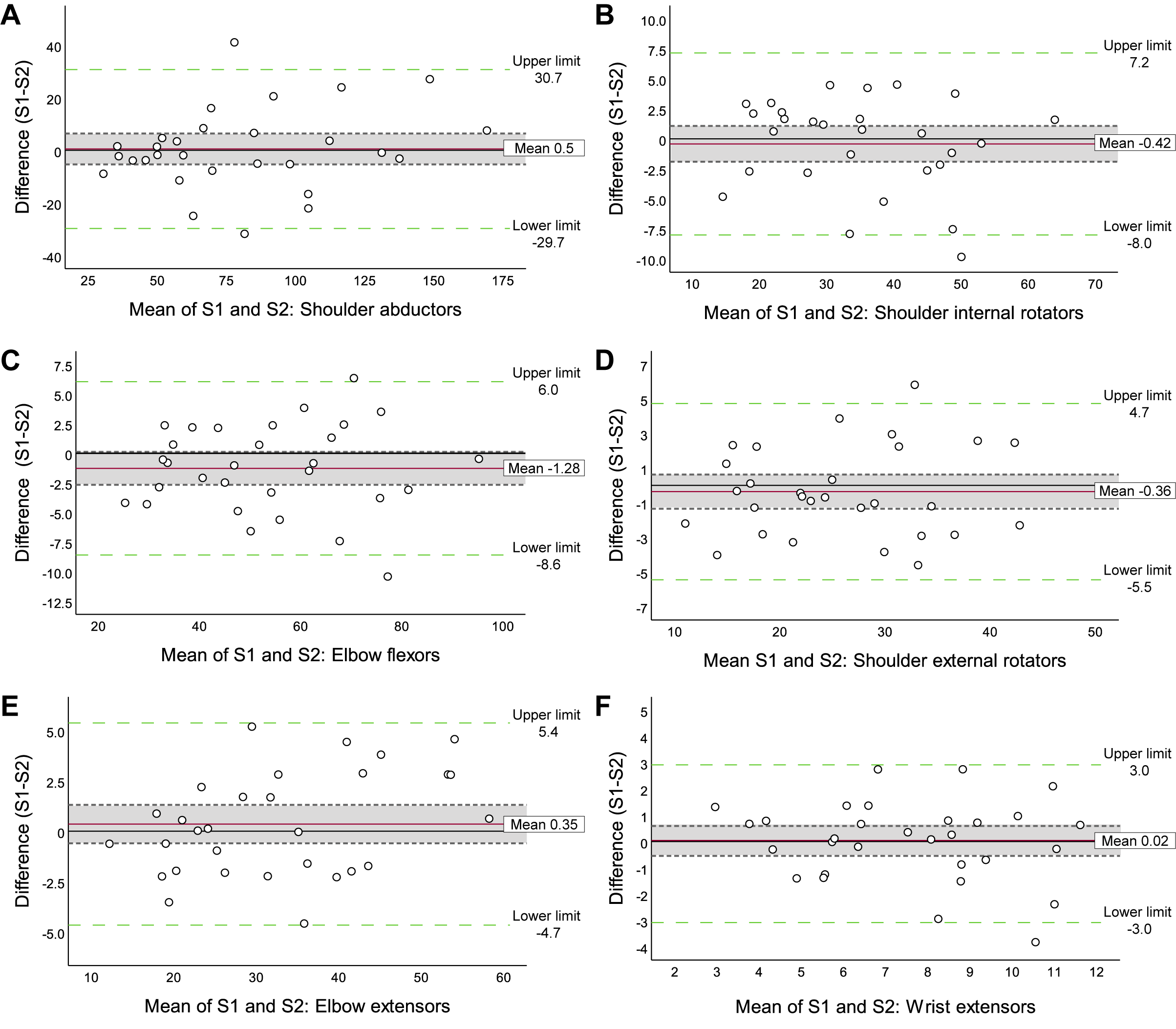

Supplement: Supplementary file 6 — Additional file 6. Bland and Altman plots, inter-rater assessment, upper limbs. Legend: Bland and Altman plots showing significant systematic bias of the mean difference of muscle torque in Nm between the first (S1) and second sessions (S2) of the shoulder abductors (A), shoulder internal rotators(B), elbow flexors (C), shoulder external rotators (D), elbow extensors (E), and wrist extensors (F). Limits of agreement (LOA) are identified by the dotted lines, from -1.96SD to +1.96SD and the mean difference by the full line in bold. The mean difference confidence intervals are depicted by the shaded area. [file 12891_2023_6400_MOESM6_ESM.tif]

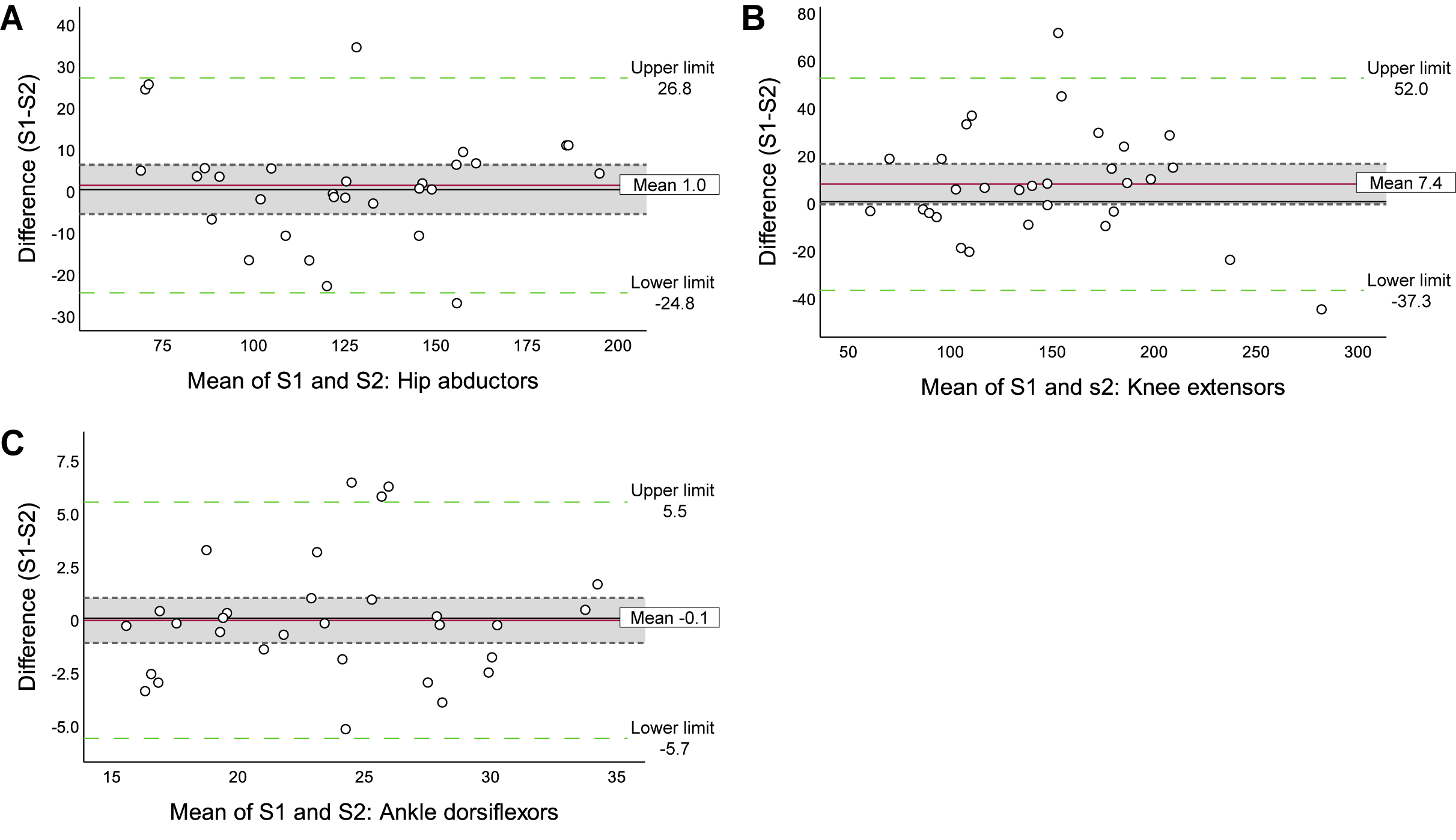

Supplement: Supplementary file 7 — Additional file 7. Bland and Altman plots, inter-rater assessment, lower limbs. Legend: Bland and Altman plots showing significant systematic bias of the mean difference of muscle torque in Nm between the first (S1) and second sessions (S2) of the hip abductors (A), knee extensors (B), and ankle dorsiflexors (C). Limits of agreement (LOA) are identified by the dotted lines, from -1.96SD to +1.96SD and the mean difference by the full line in bold. The mean difference confidence intervals are depicted by the shaded area. [file 12891_2023_6400_MOESM7_ESM.tif]
